# Supplementary material for: Factors associated with oral health care behavior of people with type 2 diabetes mellitus: A hospital-based, cross-sectional study
Source: PLoS One. 2024 May 20;19(5):e0303530. doi: 10.1371/journal.pone.0303530 (PMC11104682; doi:10.1371/journal.pone.0303530)
Supplement: S1 Checklist — (DOCX) [file pone.0303530.s001.docx]

STROBE Statement—checklist of items that should be included in reports of observational studies

|  | Item No. | Recommendation | Page  No. | Relevant text from manuscript |
| --- | --- | --- | --- | --- |
| **Title and abstract** | 1 | (*a*) Indicate the study’s design with a commonly used term in the title or the abstract | 2 | In total, 401 people with T2DM participated in the study based on their attendance at a non-communicable disease clinic at sub-district health promotion hospitals in Bueng Kan, a north-eastern province in Thailand. |
|  |  | (*b*) Provide in the abstract an informative and balanced summary of what was done and what was found | 2 | People with T2DM had good oral health status. People with T2DM with low oral health literacy, low attitude, and low level of oral health services were at a higher risk of poor oral healthcare behavior. |
| Introduction | | | |  |
| Background/rationale | 2 | Explain the scientific background and rationale for the investigation being reported | 3-4 | The prevalence rate of people with T2DM was about 10% in 2014 (8.9% for men and 10.8% for women), with the rate being higher among those with a low level of education [3]. Patients with poor glycemic control are associated with oral diseases, such as tooth decay, gingivitis, oral disease, and periodontitis. These diseases result in tooth loss, a poor digestive system, which directly impact nutrient intake. People with T2DM are more likely to have an oral disease than those without T2DM [4].  Thus, people with T2DM should be educated to carry out correct oral health care, especially those with T2DM, to encourage good oral health. |
| Objectives | 3 | State specific objectives, including any prespecified hypotheses | 4 | Hence, this study aimed to explore the factors associated with oral health care behavior among people with T2DM in Bueng Kan province, Thailand. |
| Methods | | | |  |
| Study design | 4 | Present key elements of study design early in the paper | 4 | This descriptive cross-sectional study was carried out in Bueng Kan, a north-eastern province of Thailand located 754 kilometers from Bangkok, the capital city of Thailand. |
| Setting | 5 | Describe the setting, locations, and relevant dates, including periods of recruitment, exposure, follow-up, and data collection | 4-9 | - This descriptive cross-sectional study was carried out in Bueng Kan, a north-eastern province of Thailand located 754 kilometers from Bangkok, the capital city of Thailand.  - The sample size was calculated based on a formula [14]  - the final sample size was 401 people.  - The participants were selected using a multi-stage random sampling method (stage one: cluster sampling by selecting the Bueng Khong Long district; stage two: stratified random sampling by a proportion of people with T2DM among males and females in each of the four SHPHs in the Bueng Khong Long district; and stage three: systematic random sampling using a population list in each SHPH sorted by name from which the samples were randomly selected from equal intervals to obtain the final samples. Bueng Khong Long district was randomly selected for data collection because people with T2DM in that district had similar characteristics (occupation, income, and education level) compared to the seven other districts.  - This study used a structured questionnaire to investigate factors associated with oral health care behavior of people with type 2diabetes mellitus.  - The study was approved by ethical review boards in Thailand (CSC-COA65/009). In addition, permission was sought for data collection from the Bueng Khong Long District Public Health Office.  - The researchers received a list of people with T2DM from a nurse-midwife in the SHPHs from which the samples were randomly selected and invited to participate in the study voluntarily and informed about the study before signing a consent form. Each participant answered the questionnaire for about 15–20 minutes in a room at the SHPH where they received oral health services.  - Data were analyzed using a statistical software program. Inferential statistics were conducted using regression analysis, while the dependent variable was the score for oral health care behavior.  All independent variables were included in univariate analysis for the first step. Only significant independent variables (p<0.05) from the first step were retained in the multivariate analysis based on the enter method. The degree of association was assessed using the 95% confidence interval (CI), the 0.05 significance level was used, and the coefficient of determination (R2). All independent variables were included in univariate analysis for the first step. Only significant independent variables (p<0.05) from the first step were retained in the multivariate analysis based on the enter method. The degree of association was assessed using the 95% confidence interval (CI), the 0.05 significance level was used, and the coefficient of determination (R2) An analysis of multivariable linear regression was used to describe the relationship between predictors and the oral health care behavior of people with T2DM. |
| Participants | 6 | (*a*) *Cohort study*—Give the eligibility criteria, and the sources and methods of selection of participants. Describe methods of follow-up  *Case-control study*—Give the eligibility criteria, and the sources and methods of case ascertainment and control selection. Give the rationale for the choice of cases and controls  *Cross-sectional study*—Give the eligibility criteria, and the sources and methods of selection of participants | 5 | The inclusion criteria were people with T2DM who: (1) were diagnosed with T2DM by a physician for at least one year; (2) aged 40–59 years in the year of data collection; (3) received oral services at a SHPH in Bueng Khong Long district; (4) could communicate in Thai; and (5) had ability to provide informed consent to participate in the study. |
|  |  | (*b*) *Cohort study*—For matched studies, give matching criteria and number of exposed and unexposed  *Case-control study*—For matched studies, give matching criteria and the number of controls per case | 5 | The participants were excluded if they: (1) reported having mental health problems; and (2) had serious complications (end stage of kidney disease). |
| Variables | 7 | Clearly define all outcomes, exposures, predictors, potential confounders, and effect modifiers. Give diagnostic criteria, if applicable | 8-9 | The independent variables were based on socio-demographics and oral health care of participants, oral health literacy score, score of attitudes to oral health care, oral cleaning equipment score, score for oral health services, and a score for the r-T-MSPSS. The independent and dependent variables were verified for the assumptions of the linear regression analysis based on a test of normality for the dependent variable (Z_skewness_=-0.435, Z_Kurtosis_=0.372), a test for multicollinearity problems, and the variance inflation factor. |
| Data sources/ measurement | 8* | For each variable of interest, give sources of data and details of methods of assessment (measurement). Describe comparability of assessment methods if there is more than one group | *5-9* | - The participants were selected using a multi-stage random sampling method (stage one: cluster sampling by selecting the Bueng Khong Long district; stage two: stratified random sampling by a proportion of people with T2DM among males and females in each of the four SHPHs in the Bueng Khong Long district; and stage three: systematic random sampling using a population list in each SHPH sorted by name from which the samples were randomly selected from equal intervals to obtain the final samples). Bueng Khong Long district was randomly selected for data collection because people with T2DM in that district had similar characteristics (occupation, income, and education level) compared to the seven other districts. the researchers received a list of people with T2DM from a nurse-midwife in the SHPHs from which the samples were randomly selected and invited to participate in the study voluntarily and informed about the study before signing a consent form.  - All data were entered and analyzed using a software program (Statistical Program for Social Science). Data were analyzed using a statistical software program. Descriptive statistics (such as frequency and mean) were calculated. Inferential statistics were conducted using regression analysis, while the dependent variable was the score for oral health care behavior.  The independent variables were based on socio-demographics and oral health care of participants, oral health literacy score, score of attitudes to oral health care, oral cleaning equipment score, score for oral health services, and a score for the r-T-MSPSS. All independent variables were included in univariate analysis for the first step. Only significant independent variables (p<0.05) from the first step were retained in the multivariate analysis based on the enter method. The degree of association was assessed using the 95% confidence interval (CI), the 0.05 significance level was used, and the coefficient of determination (R2) was used to describe the relationship between predictors and the oral health care behavior of people with T2DM. |
| Bias | 9 | Describe any efforts to address potential sources of bias | 12 | the research design of this study used a cross-sectional study, which might limit the causal relationship of the findings. |
| Study size | 10 | Explain how the study size was arrived at | 5 | The sample size was calculated based on a formula [14], where α=95%, acceptable margin of error (precision)=0.0692, standard deviation (SD)=0.63 [15], resulting in a minimum sample number of 351. To allow for some missing data, this study collected data from 401 people with T2DM. |

Continued on next page

| Quantitative variables | 11 | Explain how quantitative variables were handled in the analyses. If applicable, describe which groupings were chosen and why | 5  8 | The participants were selected using a multi-stage random sampling method.  The researchers received a list of people with T2DM from a nurse-midwife in the SHPHs from which the samples were randomly selected and invited to participate in the study voluntarily and informed about the study before signing a consent form. Each participant answered the questionnaire for about 15–20 minutes in a room at the SHPH where they received oral health services. |
| --- | --- | --- | --- | --- |
| Statistical methods | 12 | (*a*) Describe all statistical methods, including those used to control for confounding | 8-9 | Data were analyzed using a statistical software program. Descriptive statistics (such as frequency and mean) were calculated. Inferential statistics were conducted using regression analysis, while the dependent variable was the score for oral health care behavior. All independent variables were included in univariate analysis for the first step. Only significant independent variables (p<0.05) from the first step were retained in the multivariate analysis based on the enter method. The degree of association was assessed using the 95% confidence interval (CI), the 0.05 significance level was used, and the coefficient of determination (R2) was used to describe the relationship between predictors and the oral health care behavior of people with T2DM. |
|  |  | (*b*) Describe any methods used to examine subgroups and interactions | 9 | An analysis of multivariable linear regression was used to describe the relationship between predictors and the oral health care behavior of people with T2DM. |
|  |  | (*c*) Explain how missing data were addressed | 9 | In total, data from 401 out of 445 people with T2DM were analyzed (response rate 90.1%). |
|  |  | (*d*) *Cohort study*—If applicable, explain how loss to follow-up was addressed  *Case-control study*—If applicable, explain how matching of cases and controls was addressed  *Cross-sectional study*—If applicable, describe analytical methods taking account of sampling strategy | 9 | In total, data from 401 out of 445 people with T2DM were analyzed (response rate 90.1%). |
|  |  | (*e*) Describe any sensitivity analyses | 5-6 | A questionnaire containing VII parts was used for data collection, where the respective parts covered: (I) sociodemographic and oral health care of participants, (II) oral health literacy, (III) attitude to oral health care, (IV) oral cleaning equipment used, (V) oral health services, (VI) multi-dimensional scale of perceived social support (r-T-MSPSS), and (VII) oral health care behavior. The questionnaire was tested for validity by four Thai experts with PhD qualifications and experience in healthcare services (Structure Content Validity Index: = 0.97). A reliability test was carried out using 30 people with T2DM who did not participate in the study, with the overall Cronbach’s alpha coefficient = 0.708. |
| Results | | | | |
| Participants | 13* | (a) Report numbers of individuals at each stage of study—eg numbers potentially eligible, examined for eligibility, confirmed eligible, included in the study, completing follow-up, and analysed | 9 | In total, 401 people with T2DM participated in the study. |
|  |  | (b) Give reasons for non-participation at each stage | 9 | In total, data from 401 out of 445 people with T2DM were analyzed (response rate 90.1%). |
|  |  | (c) Consider use of a flow diagram | Not applicable | Not applicable |
| Descriptive data | 14* | (a) Give characteristics of study participants (eg demographic, clinical, social) and information on exposures and potential confounders | 9 | The majority (73.8%) were female and aged 50–59 years (72.1%). Most of the participants had completed their education to primary school level (83.8%). More than three-quarters (77.6%) had more than 20 permanent teeth and more than 4 permanent occlusal pairs (69.6%). The Decay Missing Filling Tooth (DMFT) index was 10.6 teeth/person. |
|  |  | (b) Indicate number of participants with missing data for each variable of interest | Table 1 | Table 1 Characteristics of the participants in the study (n=401) |
|  |  | (c) *Cohort study*—Summarise follow-up time (eg, average and total amount) | Not applicable | Not applicable |
| Outcome data | 15* | *Cohort study*—Report numbers of outcome events or summary measures over time | Not applicable | Not applicable- |
|  |  | *Case-control study—*Report numbers in each exposure category, or summary measures of exposure | Not applicable | Not applicable- |
|  |  | *Cross-sectional study—*Report numbers of outcome events or summary measures | *9*  *10* | -The majority of the people with T2DM had the highest means of support (S1) and were able to have an accompanying family member or friend to an oral appointment (mean=3.51, SD=0.73), followed by receptivity (R1) and being able to pay attention to oral or oral health needs (mean=3.31, SD=0.73), with the T2DM group having the lowest mean of understanding (U2) and able to read oral or oral health information brochures left in oral clinics and waiting rooms.  -The findings revealed that having the complications of diabetes mellitus (Beta=-0.097, 95%CI=-1.653, -0.046), oral health literacy (Beta=0.119, 95%CI=0.009, 0.150), educational level (Beta=0.123, 95%CI=0.103, 0.949), oral health care attitude (Beta=0.258, 95%CI=0.143, 0.333), and oral health services (Beta=0.430, 95%CI=0.298, 1.408) were statistically significant factors associated with the oral healthcare behavior of people with T2DM. The variables were able to predict 33.0% of the oral healthcare behavior of people with T2DM (R2=0.330). |
| Main results | 16 | (*a*) Give unadjusted estimates and, if applicable, confounder-adjusted estimates and their precision (eg, 95% confidence interval). Make clear which confounders were adjusted for and why they were included | 9 | All independent variables were included in univariate analysis for the first step. Only significant independent variables (p<0.05) from the first step were retained in the multivariate analysis based on the enter method. The degree of association was assessed using the 95% confidence interval (CI), the 0.05 significance level was used, and the coefficient of determination (R2) was used to describe the relationship between predictors and the oral health care behavior of people with T2DM. |
|  |  | (*b*) Report category boundaries when continuous variables were categorized | 20 | Smoking (0=no, 1=yes), Alcohol drinker (0=no, 1=yes), Complications (0=no, 1=yes), Tooth decay (0=no, 1=yes), Tooth filling (0=no, 1=yes), and Tooth missing (0=no, 1=yes). |
|  |  | (*c*) If relevant, consider translating estimates of relative risk into absolute risk for a meaningful time period | Not applicable | -Not applicable |

Continued on next page

| Other analyses | 17 | Report other analyses done—eg analyses of subgroups and interactions, and sensitivity analyses | Table 2 | Table 2 Oral health literacy of study participants (n=401). |
| --- | --- | --- | --- | --- |
| Discussion | | | | |
| Key results | 18 | Summarise key results with reference to study objectives | 10-11 | - This study focused on providing a synthesis of current evidence on the oral health services, oral health care attitudes, and oral health literacy of people with T2DM and the association of these factors with oral healthcare behavior. A large proportion of respondents rated their oral health status as tooth decay (74.6%) with an average of 7.87% having lost a tooth, which was higher than the Thai national survey of oral health in Thailand (tooth decay=49.7% and 3.2% having lost a tooth)  - In the northeastern region, 17.4% of working-age groups and 19.4% of elderly people reportedly visited oral checkup services without symptoms, which were lower proportions than overall of those seeking services for treatment [18]. People with T2DM lack access to oral services; hence, the introduction of the tele-oral system plays a role in screening. Assessment of the risk of oral diseases and early detection of diseases, such as screening for oral caries and gingivitis and examination of oral lesions by sending information to a specialist dentist for diagnosis and receiving advice, leads to the organization of disease promotion and prevention programs according to the patient’s risk [19].  - Therefore, people with T2DM should receive oral services at least once a year to effectively reduce the risk of periodontitis [23].  - Oral health literacy could contribute to increased positive oral health behavior among the participants in this study. Oral health care information access skills include mobile phone and computer applications to access information more easily, such as Facebook [26]. Channels for accessing oral health information and knowledge about oral hygiene care are necessary as a supplement to preventing oral caries and periodontal disease for the public [18], with the level of basic health literacy stemming from access to reliable and correct information. This includes understanding dentistry and having correct oral health care knowledge [27].  Therefore, oral health literacy (skills in accessing oral health information, understanding oral health care, and exchanging knowledge) might create the awareness and interest of people [28], who subsequently may make a decision to improve their oral health behavior in a sustainability manner. |
| Limitations | 19 | Discuss limitations of the study, taking into account sources of potential bias or imprecision. Discuss both direction and magnitude of any potential bias | 12 | In this study, multivariable linear regression analysis was used to adjust for confounders and to demonstrate the strengths of the association. Although this study was restricted to studying people in a government setting, most people with T2DM in Thailand still use services from government sources.. |
| Interpretation | 20 | Give a cautious overall interpretation of results considering objectives, limitations, multiplicity of analyses, results from similar studies, and other relevant evidence | 12 | Thus, the findings can be referred to other people with T2DM in similar contexts and cultures, such as agricultural areas in the Lao People’s Democratic Republic. On the other hand, the research design of this study used a cross-sectional study, which might limit the causal relationship of the findings. |
| Generalisability | 21 | Discuss the generalisability (external validity) of the study results | 12 | The value of the present study is its provision of new information regarding oral healthcare behavior, oral health status, and oral diseases. The majority of participants (people with type 2 diabetes mellitus) were female (73.8%), and 77.6% of the participants had more than 20 permanent teeth and 69.6% had four permanent occlusal pairs. The decay missing filling tooth (DMFT) index was 10.6 teeth/person. Complications from diabetes mellitus, educational level, oral health care attitude, and oral health services were statistically significant factors associated with oral healthcare behavior. People with T2DM who have low oral health literacy, low attitude, and a low level of oral health services were at a higher risk of poor oral healthcare behavior and are in need of an intervention program. |
| Other information | |  | | |
| Funding | 22 | Give the source of funding and the role of the funders for the present study and, if applicable, for the original study on which the present article is based | 12 | The authors thank all respondents who participated in this study, the Faculty of Public Health Kasetsart University for supporting resources and materials, and the government Sub-district health promotion hospitals in Bueng Khong Long, Buengkan Province, Thailand. |

*Give information separately for cases and controls in case-control studies and, if applicable, for exposed and unexposed groups in cohort and cross-sectional studies.

**Note:** An Explanation and Elaboration article discusses each checklist item and gives methodological background and published examples of transparent reporting. The STROBE checklist is best used in conjunction with this article (freely available on the Web sites of PLoS Medicine at http://www.plosmedicine.org/, Annals of Internal Medicine at http://www.annals.org/, and Epidemiology at http://www.epidem.com/). Information on the STROBE Initiative is available at www.strobe-statement.org.
