# Supplementary figures and images for: Factors associated with oral health care behavior of people with type 2 diabetes mellitus: A hospital-based, cross-sectional study
Source: PLoS One. 2024 May 20;19(5):e0303530. doi: 10.1371/journal.pone.0303530 (PMC11104682; doi:10.1371/journal.pone.0303530)

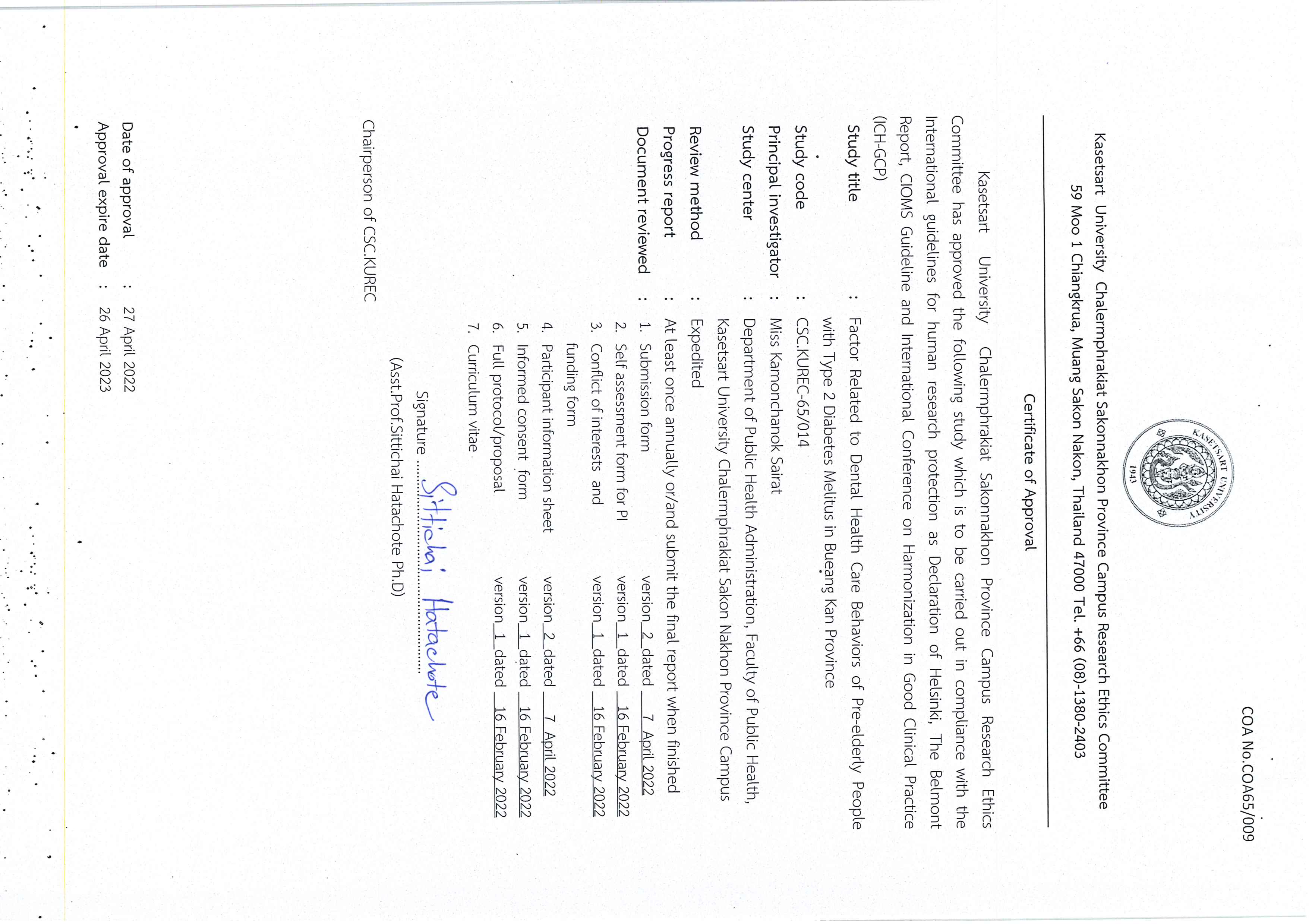

Supplement: S1 File — (JPG) [file pone.0303530.s003.jpg]
