# Supplementary material for: Factors associated with oral health care behavior of people with type 2 diabetes mellitus: A hospital-based, cross-sectional study
Source: PLoS One. 2024 May 20;19(5):e0303530. doi: 10.1371/journal.pone.0303530 (PMC11104682; doi:10.1371/journal.pone.0303530)
Supplement: Attachment — Submitted filename: PLOS 2024 thai.pdf [file pone.0303530.s004.pdf]

PLOS ONE [PONE-D-23-32407]

Factors associated with oral health care behavior of people with type 2 diabetes mellitus: A hospital-based, cross-sectional study

items	Comment
Page 9/31, last para, second last line<br><br>In Health Region 8, the incident rates of diabetes per 100,000 population in for the period 2018, 2019, 2020, and 2021 were 511.53, 525.28, 580.10, and 538.65, respectively	editorial – delete "jn"
Page10/31 line 3 -6<br><br>A screening protocol for oral health for people with T2DM aged 45–59 years showed that 19.43%, 17.15%, and 11.21% of the people were screened in the fiscal years 2020, 2021, and 2022, respectively. *In addition, 16.03%, 15.84%, and 12.11%, respectively, of the people received oral health examinations*	vague statement related to statement above. Are investigators trying to say " Of the above. 16.03% etc… ? then Replace or delete "In addition"
Page 12/31 second last line.. A questionnaire containing VII parts was	Replace VII with words - "seven"
Page 15/31 on Table 1 **Results** line 2<br>*The majority (73.8%) were female and aged 50–59 years (72.1%)*	Confusing interpretation of 1st para on RESULTS. The 73.8% is related to proportion of women in study from overall total N=401?..<br>The figure 72.1% is the proportion of those *aged 50–59 years (72.1%) from N=401?? Not from the above female 73.8% right ?*<br><br>*Need to rewrite* eg. Overall, majority of the study participants were females (73.8%), 50-59 years old (72/1%), Married (79.3%) etc…
Page 15/31 para 1 under Results and also pages 24-26/31 Table 1 presentation	Table 1 (3 pages) appear to present 3 domains, namely; sociodemographic profile, general health and oral health status. Each has its own set of variables. But they were incomplete and poorly described or not included in the Result section write up<br>For example:<br>1. There are many sociodemographic information in Table 1 presented. But authors only describe selectively, namely on 4 variables gender, age, education and marital status. How about the others? If not important or meaningful to

	study the outcome, then do not include in Table 1 presentation.<br><br>But from my understanding of the analysis section, all variables were put into a stepwise regression model. Therefore authors should at least provide a general but complete description of Table 1 **sociodemographic** variables. I also did not find any description of the related health and social habits information, yet it was collected in the questionnaire and presented in Table 1.<br><br>The third domain is on oral health status. As described in methodology these were not collected by the investigators, they were secondary data obtained from patient records and done by the district office. (*the medical records of the SHPH, such as oral health status (number of permanent teeth, number of occlusal pairs, tooth decay, teeth missing,teeth filling,* Pg 14/31 1st para)<br><br>2. Should it be presented in Table 1 all jumbled up?? Furthermore there is a need to acknowledge these data with at least an asterik and a note below the table of the source.<br><br>Suggest to split Table 1 into at least 2 tables. One focusing on sociodemographic, socio-cultrual practices & general health overview and second on oral health profile info,
Page 15/31 last Para, description/interpretation of Table 2	Results description needs to improve as well. Delete note at bottom of table on SD.
Page 16/31 first para describing Table 3<br>The findings revealed that having the complications of diabetes mellitus (Beta=- 0.097, 95%CI=-1.653, -0.046), oral health literacy (Beta=0.119, 95%CI=0.009, 0.150), educational level (Beta=0.123, 95%CI=0.103, 0.949), oral health care attitude (Beta=0.258, 95%CI=0.143, 0.333), and oral health services (Beta=0.430, 95%CI=0.298, 1.408) were statistically significant factors associated with the oral healthcare behavior of people with T2DM	Result's descriptions on Table 3 needs rephrasing. Variables affecting OHCare behaviors mentioned OH literacy good or poor??), educ level (high or low?) etc
Page 17/31 Open statement in Discussion is<br>*This study focused on providing a synthesis of current evidence on*	P9 10/31 Stated Study Objective<br>*Hence, this study aimed to explore the factors associated with oral health care behavior among people with T2DM in Bueng…*

the oral health services, oral health care attitudes, and oral health literacy of people with T2DM and the association of these factors with oral healthcare behavior	Maybe related but vague. Needs to answer stated objectives . Maybe need to expand sub-variables related to oral health behavior?
