# Supplementary material for: Factors associated with oral health care behavior of people with type 2 diabetes mellitus: A hospital-based, cross-sectional study
Source: PLoS One. 2024 May 20;19(5):e0303530. doi: 10.1371/journal.pone.0303530 (PMC11104682; doi:10.1371/journal.pone.0303530)
Supplement: Attachment — Submitted filename: responses to revewers 28032024.docx [file pone.0303530.s005.docx]

**Response to reviewers' comments**

**Dear Editor,**

We thank you and the reviewers for remarks which have helped us improve the manuscript. Please see our response below.

Reviewer #1: In the manuscript entitled: "Factors associated with oral health care behavior of people with type 2 diabetes mellitus: A hospital-based, cross-sectional study" the authors aimed to assess the factors associated with oral healthcare behavior among people with T2DM in Thailand. The authors found that the majority of participants were female (73.8%) and most of the participants had received primary education (83.8%). More than three-quarters (77.6%) had more than 20 permanent teeth, while many of the participants (69.6%) had four permanent occlusal pairs. A decay missing filling tooth index was 10.6 teeth/person. The authors concluded that people with T2DM had good oral health status. People with T2DM with low oral health literacy, low attitude, and low level of oral health services were at a higher risk of poor oral healthcare behavior.

Major comments: In general, the idea and innovation of this study regards the analysis of oral health in patients with diabetes is interesting and novel because the role these aspects in medicine are validated but further studies on this topic could be an innovative issue in this field could be open a creative matter of debate in literature by adding new information. Moreover, there are few reports in the literature that studied this interesting topic with this kind of study design. The study was well conducted by the authors; However, there are some concerns to revise that are described below.

The introduction section resumes the existing knowledge regarding the important factor linked with the impact mediators involved together oral health and with periodontitis. However, as the importance of the topic, the reviewer strongly recommends, before a further re-evaluation of the manuscript, to update the literature through read, discuss and must cites in the references with great attention all of those recent interesting articles, that helps the authors to better introduce and discuss the role of mediators (NT-PRO-BNP and TGF beta 1) in periodontitis and related recessions by adding as a references these article, before any further assessment of the manuscript: 1) DOI: doi: 10.1002/JPER.23-0063. PMID: 37433155; 2) DOI: 10.1177/1721727X1301100217

Response: Thank you for your suggestion and comments. We have read the articles and written texts in the Introduction section, page 3/25 and added in the reference list.

The authors should be better specified, at the end of the introduction section, the rationale of the study and the aim of the study.

**Response:** We have already specified the rationale and aim of the study in the introduction section, page 4/25 and in the Aim section page 5/25.

In the central section, should better clarify inclusions and exclusions criteria of the selected sample.

**Response:** We have already clarified the criteria in the Participants section, page 6/25.

Please better state the results obtained in the abstract.

**Response:** We have already rewritten and added texts for the results in the Results section, pages 9-10/25.

The discussion section appears well organized with the relevant paper that support the conclusions, even if the authors should better discuss the relationship regarding the by periodontitis in and risk of oxidative stress evolution that could improve the quality of life in periodontitis patients which undergo diabetes.

**Response:** We have already rewritten and added texts in the Discussion section, page 12/25.

The conclusion should reinforce in light of the discussions.

**Response:** We have already rewritten and added texts in the Conclusions section, page 13/25.

Minor Comments:

Abstract:

- Better formulate the abstract section by better describing the aim of the study

**Response:** We have already rewritten the aim of the study in the Abstract section, page 2/25.

Discussion

- Please add a specific sentence that clarifies the results obtained in the first part of the discussion

**Response:** We have already added texts in the Discussion section, page11/25.

Reviewer #2: Manuscript Number: PONE-D-23-32407

Full Title: Factors associated with oral health care behaviour of people with T2DM

Dear Authors. Overall, I think it is a good article that needs a few changes or additions (See below). I would suggest accepting with minor revisions.

Methods

1. Authors should clearly define what they mean by 'oral healthcare behavior.' (e.g. the actions and habits individuals adopt....).

**Response:** We have already added texts to clarify this in the Introduction section, page 4/25.

2. Authors should mention and describe their cross-sectional design method somewhere in the Methods, preferably at the start (e.g., in a design section).

**Response:** We have already added texts for the design of the study in the Methods section, page 5/25.

Discussion

1. Authors should consider providing suggestions for future research based on gaps they identified, and their limitations.

**Response:** We have already added texts to suggest for future research in the Strengths and limitations section, page 13/25.

Conclusions

1. The authors should also expand on what they mean by 'intervention program'.

**Response:** We have already added texts in the Conclusions section, page 13/25.

Reviewer #3: A relevant and important study as it relates to oral health and systemic disease (DM) especially among LMIC and MMIC. will certainly add to the current scarcity of information on these issues in the Asian environment particularly. Methodology well described. Concern is on data presentation and interpretation of Results especially Table 1. subsequently needs to look into refinement of discussion. Conclusion acceptable. Actually Abstract is better written.

**Response:** Thank you for your comments. We have rewritten results accordingly in the Results section, pages 9-10/25 and in the Abstract section page 2/25.

PLOS ONE [PONE-D-23-32407]

**Factors associated with oral health care behavior of people with type 2 diabetes mellitus: A hospital-based, cross-sectional study**

| items | Comment | Response |
| --- | --- | --- |
| Page 9/31, last para, second last line  In Health Region 8, the incident rates of diabetes per 100,000 population in for the period 2018, 2019, 2020, and 2021 were  511.53, 525.28, 580.10, and  538.65, respectively | editorial – delete “jn” | We have deleted “in” in the Introduction section, page 4/25. |
| Page10/31 line 3 -6  A screening protocol for oral health for people with T2DM aged 45–59 years showed that 19.43%, 17.15%, and 11.21% of  the people were screened in the fiscal years 2020, 2021, and 2022, respectively. *In addition, 16.03%, 15.84%, and 12.11%, respectively, of the people received oral health examinations* | vague statement related to statement above. Are investigators trying to say “ Of the above. 16.03% etc… ? then Replace or delete “In addition” | We have deleted “In addition” and “Of the above. 16.03% etc.… ? in the Introduction section, page 4/25. |
| Page 12/31 second last line.. A questionnaire containing VII parts  was | Replace VII with words - “seven” | We have replaced VII with words - “seven” in the Instrument section, page 6/24. |
| Page 15/31 on Table 1 **Results**  line 2  *The majority (73.8%) were female and aged 50–59 years (72.1%)* | Confusing interpretation of 1^st^ para on RESULTS. The 73.8% is related to proportion of women in study from overall total N=401?..  The figure 72.1% is the proportion of those *aged 50–59 years (72.1%) from N=401?? Not from the above female 73.8% right*  *?*  *Need to rewrite* eg. Overall, majority of the study participants  were females (73.8%), 50-59 years old (72/1%), Married (79.3%) etc… | Thank you for your comments. We have already rewritten and added texts for the results in the Results section, pages 9-10/25. |
| Page 15/31 para 1 under Results and also pages 24-26/31 Table 1 presentation | Table 1 (3 pages) appear to present 3 domains, namely; sociodemographic profile, general health and oral health status. Each has its own set of variables. But they were incomplete and poorly described or not included in the Result section write up  For example:  1. There are many sociodemographic information in Table 1 presented. But authors only describe selectively, namely on 4 variables gender, age, education and marital status.  How about the others? If not important or meaningful to study the outcome, then do not include in Table 1 presentation.  But from my understanding of the analysis section, all variables were put into a stepwise regression model. Therefore authors should at least provide a general but complete description of Table 1 sociodemographic variables. I also did not find any description of the related health and social habits information, yet it was collected in the questionnaire and presented in Table 1.  The third domain is on oral health status. As described in methodology these were not collected by the investigators, they were secondary data obtained from patient records and done by the district office. (the medical records of the SHPH, such as oral health status (number of permanent teeth, number of occlusal pairs, tooth decay, teeth missing,teeth filling, Pg 14/31 1st para)  2. Should it be presented in Table 1 all jumbled up?? Furthermore there is a need to acknowledge these data with at least an asterik and a note below the table of the source.  Suggest to split Table 1 into at least 2 tables. One focusing on sociodemographic, socio-cultrual practices & general health  overview and second on oral health profile info, | Thank you for your comments. We have already rewritten and added texts in the Table 1 section, pages 20-22/25. |
| Page 15/31 last Para, description/interpretation of  Table 2 | Results description needs to improve as well. Delete note at bottom of table on SD. | Thank you for your comments.  We have improved the results in the Result section. However, we remain provide full name of SD in the bottom of the table, page 20/25. |
| Page 16/31 first para describing Table 3  The findings revealed that having the complications of diabetes mellitus (Beta=- 0.097, 95%CI=- 1.653, -0.046), oral health literacy (Beta=0.119, 95%CI=0.009, 0.150), educational level (Beta=0.123, 95%CI=0.103,  0.949), oral health care attitude (Beta=0.258, 95%CI=0.143,  0.333), and oral health services (Beta=0.430, 95%CI=0.298,  1.408) were statistically significant factors associated with the oral healthcare behavior of people with T2DM | Result’s descriptions on Table 3 needs rephrasing.  Variables affecting OHCare behaviors mentioned OH literacy good or poor??), educ level (high or low?) etc | Thank you for your comments.  We used oral health literacy as a score (continuous variable) and put in the analysis. Therefore, we did not categorise the score into good/poor level. |
| Page 17/31 Open statement in Discussion is  This study focused on providing a  synthesis of current evidence on | P9 10/31 Stated Study Objective  Hence, this study aimed to explore the factors associated with oral health care behavior among people with T2DM in  Bueng… | We have already added texts in the Discussion section, page11/25. |
